# A deepfake-based study on facial expressiveness and social outcomes

**DOI:** 10.1038/s41598-024-53475-5

**Published:** 2024-02-13

**Authors:** Laetitia A. Renier, Kumar Shubham, Rahil Satyanarayan Vijay, Swasti Shreya Mishra, Emmanuelle P. Kleinlogel, Dinesh Babu Jayagopi, Marianne Schmid Mast

**Affiliations:** 1 Department of Organizational Behavior, Faculty of Business and Economics (HEC), University of Lausanne, Internef #558, 1015 Lausanne, Switzerland; 2 International Institute of Information Technology, Bangalore, India; 3 CEMOI Laboratory, IAE Reunion, University of Reunion Island, Saint Denis, France

**Keywords:** Social behaviour, Computer science

## Abstract

Artificial intelligence (AI)-generated media is used in entertainment, art, education, and marketing. AI-generated faces or facial expressions using deepfake-based technologies might also contribute to nonverbal behavior studies. As a proof of concept, in this research, we test the replicability of past results regarding the positive effects of facial expressiveness (e.g., gazing, nodding, and smiling) on social outcomes. Because the facial expressions when listening to someone (i.e., nonverbal immediacy) encompass several behaviors that are typically related (i.e., smiling while nodding), the effect of combinations of these behaviors is challenging to study. We thus test the effect of facial expressiveness (gazing, nodding, and smiling vs. none) on first impression judgements in a job interview setting using AI-generated experimental video material. We measure how competent, warm, and favorably independent observers (*n* = 823) perceive the targets (*n* = 159) showing AI-generated facial expressions. Our results replicate past research showing that the more targets are facially expressive, the more they are perceived favorably. Apart from supporting evidence of the importance of facial expressiveness for conveying a positive impression, we show the benefits of relying on AI-generated experimental video material for the study of nonverbal behavior.

## Introduction

Machine learning generative models allow to create a new synthetic image or video of an individual while controlling for their appearance or actions^1,2^. One AI-based techonology, namely deepfake, enables users to swap faces or control facial expressions to make people seemingly act in authentic ways, in events that have not actually taken place in reality. Deepfake is well known for its use in entertainment, art, and culture (e.g., listening to and taking selfies with Dalí)^3^, or advertising^4^ as well as for its misuse in generating controversial content (e.g., fake news, pornographic content)^5,6^. Advances in these technologies, rendering “AI-generated characters”^7^ and content more and more realistic, make it thus more difficult to distinguish fake from truthful content^8^.

While this poses a threat to society^6^, deepfake offers an increased control over content and realism that can be of added-value for experimental and psychological research^9,10^. For instance, others have discussed the use of deepfake for the study of human behaviors (e.g., video corpus development^10^, negotiation studies^11^), to support learning and well-being^7^, or to protect personal identity (deidentification of patients^12,13^).

In this research, we show how deepfake can be used to overcome methodological shortcomings regarding the study of facial expressiveness (e.g., gazing, nodding, and smiling) and its effect on observers. Traditionally, studies on the effect of facial expressiveness on observers use video excerpts of people (targets as either actors or untrained participants) who vary in expressiveness and ask observers about their impressions. For example, research shows that people who exhibit more facial expressiveness (e.g., gazing, nodding, and smiling) are liked more and perceived more favorably (see next section for a review). While real facial expressions have high ecological validity, they pose problems in the sense that nonverbal behaviors show idiosyncratic variations (e.g., a person’s smile might be broader or more subtle than another person’s smile) and many of the nonverbal expressive behaviors are related to other target characteristics, such as women being more nonverbally expressive than men^14^. To disentangle the effect of gender from the effect of the nonverbal behavior, researchers need to be able to separate them (e.g., have a woman’s face and a man’s face smiling or gazing exactly in the same way in terms of intensity and frequency). Using AI-generated facial expressions allows for the standardization of the expressive behavior of any individual with respect to temporality (e.g., when and for how long one makes eye contact), frequency (smiling two vs. five times), intensity (e.g., slight vs. frank nods), or co-occurrence (e.g., smiling when nodding), irrespective of gender, age, ethnicity, or other facial appearance factors (e.g., attractiveness, hairstyle, facial marks). Using AI-generated characters as targets in videos allows researchers to disentangle facial appearance cues (e.g., gender, skin color, hairstyle, age, attractiveness) from facial behaviors and to test how they independently affect social interaction outcomes. When attempting to understand the impact of nonverbal behaviors on social outcomes, it is important to disentangle facial appearance from facial expressiveness. This is because these two variables should not be correlated, otherwise, causal claims are not warranted. In other words, we circumvent endogeneity issues^15,16^.

We use AI-generated videos that use portrait-like picture of individuals and animate it with expressive facial behavior thanks to computer vision and machine learning^17,18^. Using such AI-generated videos is resource-conserving because once the algorithms have been developed, researchers are able to scale up their experiment because a huge number of photos can be animated easily and quickly.

We thus suggest that the main advantages of this AI-based approach is that it makes it possible to study the effect of nonverbal behavior on social outcomes while (a) controlling for characteristics of the person expressing these behaviors, (b) standardizing temporality, intensity, frequency, and co-occurrence of the behaviors in a controlled way, and (c) reducing costs associated with the development of the experimental video material.

As a validity check of the use of AI-generated video stimuli, we aimed to replicate past results showing that facial expressiveness is related to more favorable first impressions. We therefore showed AI-generated videos of the same people, called *targets*, with either an expressive (gazing, nodding, and smiling) or an non-expressive face (looking away, no nodding, and no smiling) to external judges, called *observers*. We expected that the expressive targets would elicit more positive social outcomes (i.e., perception of competence, warmth, and overall favorable impression based on perception of hireability, impression, and skills) than the non-expressive targets.

### Expressiveness in job interviews

Facial expressiveness when listening to an interaction partner is often coined in the field of nonverbal behavior as immediacy, defined as “communication behaviors that enhance closeness to and non-verbal interaction with another”^19^ and it refers to behavior reducing physical and/or psychological distance^20^. More recent definitions have proposed that immediacy refers to the degree to which individuals appear involved in an interaction or to a set of behaviors characterizing approach tendencies without particularly reflecting positive affect^21^. Overall, immediacy is often operationalized as facial expressiveness (e.g., gazing, nodding, smiling) manifested while listening to someone. It signals closeness and involvement which subsequently translate into positive impressions.

Several theories and models explain how expressive behavior translates into social interaction outcomes. Based on the dual process theory, the said behaviors influence hiring decisions through System 1, characterized by automatic, non-conscious, and quick decisions^22^. Expressive nonverbal cues are thus (a) readily available and easily processed (*initial impression formation*), (b) evoke affective responses such as similarity and liking (*affect/immediacy heuristic*), and (c) are used to infer interviewee personality and competence (*dispositional attribution*). Two additional mechanisms have been suggested^23^: the *salience hypothesis*, positing that expressive nonverbal behavior helps recruiters differentiate interviewees when interviewees are perceived as very similar; the *reinforcement theory*, stating that after the initial impression formation, recruiters reinforce the said impression based on interviewee nonverbal behavior, which in return fosters interviewee nonverbal behaviors confirming the recruiter’s initial impression. Taken together, these theories suggest that being expressive is automatically associated with favorable outcomes, such as being liked by the perceivers. This suggests that being expressive offers an advantage during social interactions.

Nonverbal immediacy behaviors are associated with positive interpersonal outcomes. In the field of education, teachers showing nonverbal expressiveness are considered rewarding^24^, motivating^25^, or as reducing stress for their students^26^. In the field of personnel selection and particularly job interviews, nonverbal immediacy behaviors are associated with more favorable first impressions^23^ and more positive outcomes (e.g., hireability and performance)^27^. Smiling, eye contact, gestures, proxemic (interpersonal distance), attentive posture, and body orientation were all positively related to positive ratings of interviewees (e.g., competence, the likelihood of acceptance and of success, motivation)^28^. Gazing, smiling, hand gestures, forward-leaning posture, and attractiveness had a positive impact on interviewer impressions of interviewees in terms of competence and performance^29^. Additionally, combining these behaviors might be even more beneficial: Interviewees showing more nonverbal immediacy cues (e.g., more gazing, nodding, smiling, open and forward-leaning, and hand gestures) were rated more favorably^30^.

Specific facial expressive cues affect interview outcomes differently. For instance, interviewees showing a normal or high level of *gazing* at the interaction partner were rated as more hireable and more credible^31^, as well as more competent^32^. Concerning *nodding* while listening only, research shows that nodding while listening did not have a significant effect on the outcome^33^. Concerning *smiling* only, authentic smiles (vs. fake or managed smiles) are associated with more positive outcomes (e.g., employment decisions) using both synthetic and human faces^34^. Hireability scores are also higher when interviewees smiled less in the middle of the interview compared to the beginning and the end of the interview^35^. When comparing gazing, nodding, and smiling, a meta-analysis^22^ showed that gazing had the strongest association with positive interview outcomes, and nodding came in second position. It did not confirm the positive effect of smiling on interview outcomes.

The effect of expressive nonverbal behaviors on interview outcomes is moderated by *interview characteristics* such that research finds a greater effect in unstructured compared to structured interviews^36^, by *job characteristics* such that gazing at the interviewer was more important (positive effect) when applying for a high-status job than a low-status job^37^ or that smiling in the case of jobs for which smiling less is expected is detrimental^35^, and by *individual characteristics* such that expressed nonverbal behaviors during the job interview appear to play a greater role for interview outcomes among women than men for most cues^22^. Furthermore, judges’ decisions might be driven by their gender expectations^22,35,38^. These findings suggest that it is crucial to control for these characteristics in order to accurately assess the effect of facial expressiveness on job interview outcomes.

### The current study

To test the effect of showing a combination of facial expressiveness cues (gazing, nodding, and smiling vs. showing none) on first impression judgements, we employ an AI-based technology (i.e., deepfake) to develop our target video stimuli. Instead of relying on video recordings of targets enacting behaviors (called the “traditional approach” here and in^39^), we relied on portrait-like pictures of individuals (target selfies) and used feature extraction and machine learning to generate videos from those pictures showing a predefined and constant set of facial expressiveness cues. This enables us to rely on highly standardized and, at the same time, naturalistic experimental material to study expressive facial behaviors.

To create AI-generated characters, two sets of input are required using one-shot deepfake (i.e., generation of videos using one unique picture of the target)^40^: (a) the *target input* which is a frontal portrait picture of the target (e.g., selfie) in front of a neutral background (the greater the contrast, the better the quality of the synthesized video) depicting a well-lit face and the most neutral facial expression possible (expression or shadows on the face might affect the quality of the synthesized video) and (b) the *referent input* which consists of a video of an individual showing the facial expressiveness cues to put on the target selfie (target input) to animate the photo and thus generate the video stimuli. To do so, the algorithm extracts the cues from the referent video and transposes them onto the target picture thus generating videos of the target showing an animated face (generated *output*). It is noteworthy that this procedure creates a video of a person solely based on that person’s portrait-like picture.

To study the effect of facial expressiveness on interview outcomes, we focused on gazing, nodding, and smiling for two reasons. First, research has shown that the said nonverbal behaviors have an impact on social interaction outcomes (see previous section) and are relevant in the job interview context. Second, focusing on facial expressiveness meant relying on one-shot deepfake. While this choice of technology offers greater visual and animation quality of the generated faces than technologies transferring body motions (see, for example, the use of posetransfer^41^), we had to focus on nonverbal behaviors manifested in the head/face region and thus keep a selfie view in the generated videos.

In this study, we asked 159 targets to provide a selfie to generate short videos showing the targets being facially expressive (gazing, nodding, and smiling while listening to an interview question) or not. We then asked observers to watch the target videos (one video per condition) and to report their perception of the targets on competence, warmth, and overall favorable impression. This allows to test whether expressive targets are perceived as more competent, warm, and favorably than non-expressive targets (Hypothesis 1). Then, because the literature suggests that individual characteristics might affect said effects, we study the effect of being facially expressive on social outcomes above and beyond target culture and target gender (Hypothesis 2). To do so, our sample of targets are composed of Swiss and Indian, female and male, targets. Further details on material development (i.e., deepfake generated videos) and data collection are provided under the Method section.

## Results

Table 1 presents the descriptive statistics and correlation matrix for the main variables across conditions at the target level (*N* = 159). Before testing our hypotheses, we first performed one-sided one-sample *t*-tests testing the perception of realness of the target videos to ensure that the generated videos were perceived as sufficiently realistic. These analyses serve as a quality check of the target videos. Results showed that observers perceived the videos as realistic. Perception of realness was statistically significantly higher than the scale mid-point (3), assuming that a score of 3 or higher means that the videos were perceived as sufficiently realistic in each condition: facially expressive, *M(SD)* = 3.41(0.91), *t*(822) = 12.83, *p* < 0.001, 95% CI [0.38, 0.52], Cohen’s *d* = 0.45; facially non-expressive, *M(SD)* = 3.08(1.00), *t*(822) = 2.16, *p* = 0.031, 95% CI [0.01, 0.14], Cohen’s *d* = 0.08. This is consistent with research showing that it is difficult to differentiate generated videos from real video-recordings of people^40,42^.

**Table 1:** Descriptive statistics and correlation matrix for the main variables across conditions at target level (*N* = 159).

		*M*	*SD*	1	2	3	4	5	6	7	8	9	10	11
1	P. of competence (expressive)	3.54	0.29											
2	P. of competence (non-expressive)	2.79	0.31	0.06										
3	P. of warmth (expressive)	3.55	0.29	0.64 **	0.15 ^+^									
4	P. of warmth (non-expressive)	2.81	0.34	0.03	0.68 **	0.17 *								
5	P. of overall fav. impression (expressive)	3.59	0.35	0.83 **	0.11	0.70 **	0.01							
6	P. of overall fav. impression (non-expressive)	2.31	0.43	0.07	0.77 **	0.19 *	0.76 **	0.09						
7	P. of hireability (expressive)	3.35	0.38	0.83 **	0.09	0.65 **	– 0.01	0.94 **	0.05					
8	P. of hireability (non-expressive)	2.29	0.42	0.09	0.74 **	0.17 *	0.70 **	0.10	0.95 **	0.07				
9	P. of impression (expressive)	3.63	0.38	0.82 **	0.04	0.68 **	– 0.03	0.94 **	0.04	0.85 **	0.06			
10	P. of impression (non-expressive)	2.36	0.46	0.08	0.78 **	0.18 *	0.73 **	0.11	0.96 **	0.07	0.90 **	0.07		
11	P. of skills (expressive)	3.80	0.37	0.66 **	0.17 *	0.61 **	0.07	0.90 **	0.16 *	0.75 **	0.14 ^+^	0.74 **	0.16 *	
12	P. of skills (non-expressive)	2.26	0.47	0.03	0.66 **	0.18 *	0.73 **	0.05	0.93 **	0.01	0.82 **	– 0.02	0.82 **	0.15 ^+^

*Notes*. + *p* < 0.10, * *p* < 0.05, ** *p* < 0.01.

To test the first hypothesis, namely that being facially expressive (i.e., gazing, smiling, and nodding while listening to a social interaction partner) results in better interaction outcomes than being facially non-expressive (i.e., looking away without nodding and without smiling), we calculated three paired *t*-tests, one per dependent variable. We made these analyses at the observer level (*N* = 823).

**Figure 1:**
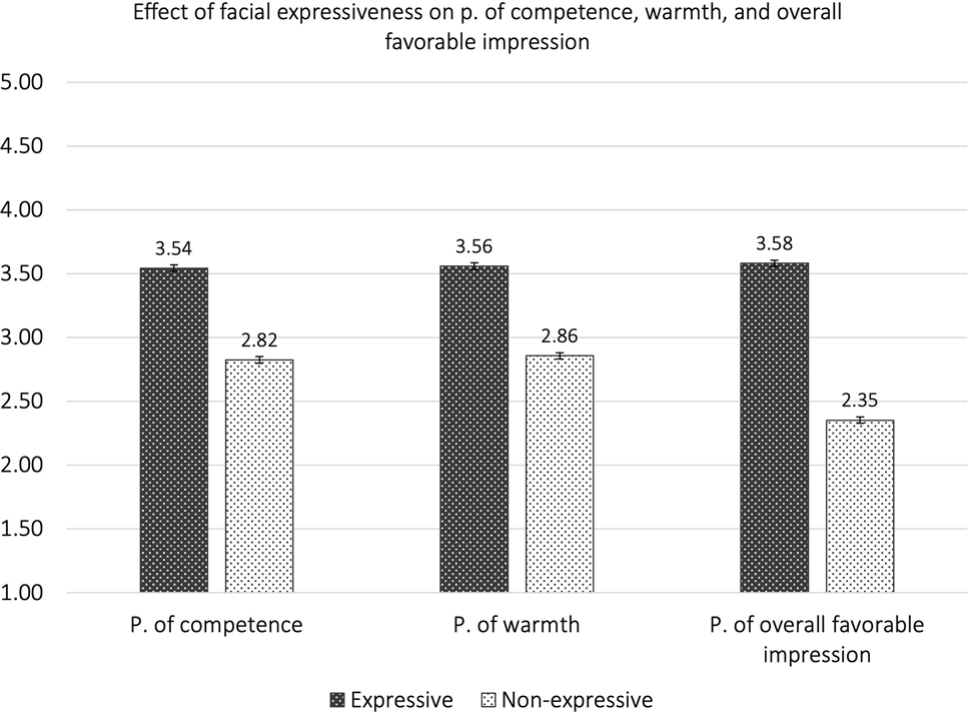
Effect of facial expressiveness on perception of competence, warmth, and overall favorable impression.

Results confirmed Hypothesis 1 and showed that facially expressive targets were perceived as more competent [*M(SD)* = 3.54(0.56), *t*(822) = 25.82], warmer [*M(SD)* = 3.56(0.58), *t*(822) = 24.98], and made a more favorable overall impression [*M(SD)* = 3.54(0.68),* t*(822) = 32.25] than non-expressive targets [perception of competence: *M(SD)* = 2.80(0.61); perception of warmth: *M(SD)* = 2.82(0.65); perception of overall favorable impression: *M(SD)* = 2.32(0.79)]. All results were statistically significant and remained significant after applying Bonferroni correction for three comparisons (corrected α = 0.017) to avoid Type I error, all *p*s < 0.001. The effect sizes are large: perception of competence: 95% CI [0.82, 0.98], Cohen’s *d* = 0.90; perception of warmth: 95% CI [0.79, 0.95], Cohen’s *d* = 0.87; perception of overall favorable impression: 95% CI [1.04, 1.21], Cohen’s *d* = 1.12 (see Fig. 1).

We additionally present, under Appendix 1, the results of non-parametric tests because most of the dependent variables were not normally distributed at the observer level. Results of non-parametric tests are considered in this research as a robustness check for the main results (i.e., perception of realness and the effect of facial expressiveness only on the dependent variables). These results showed similar effects.

**Figure 2:**
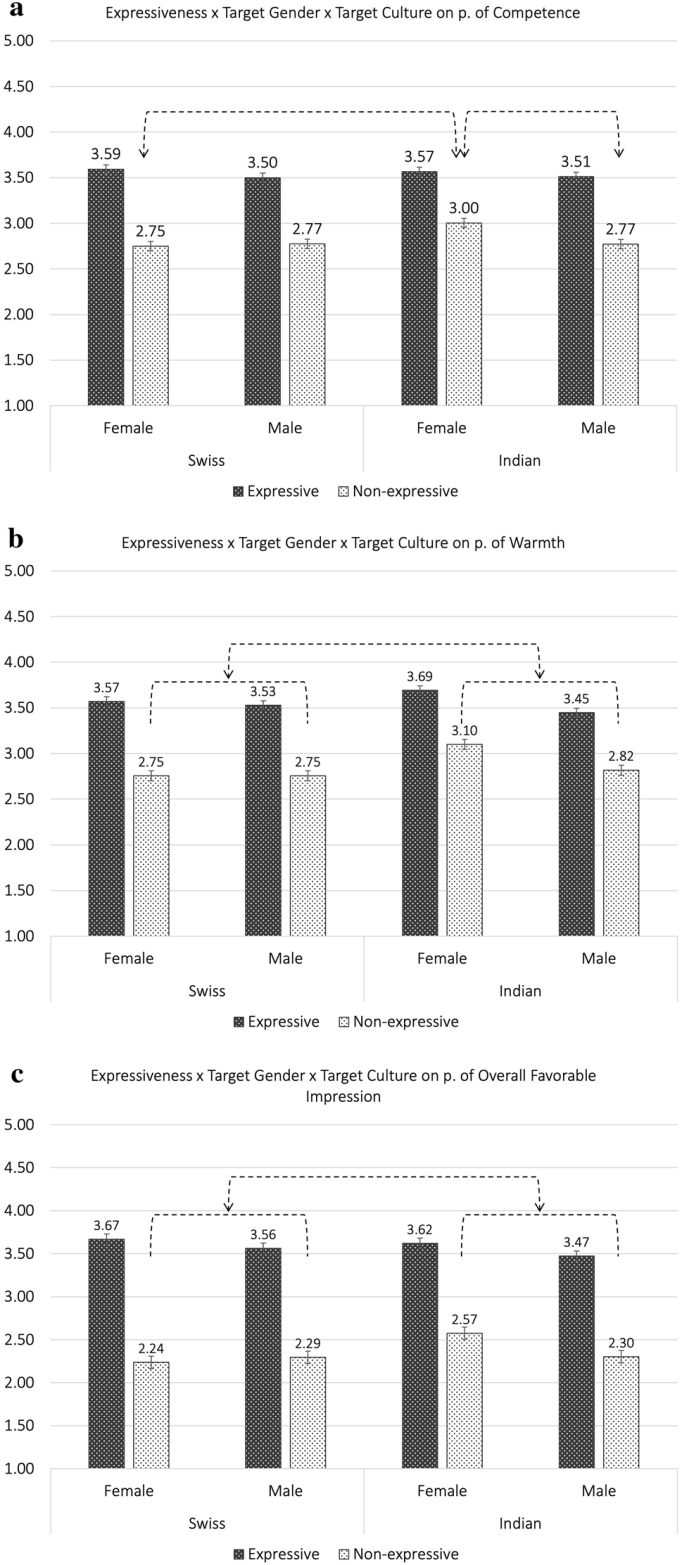
Effect of facial expressiveness by target gender by target culture on perception of competence, warmth, and overall favorable impression. (**a**) Effect of facial expressiveness by target gender by target culture on perception competence. *Note*. The interaction effects are depicted using the dashed lines and arrows. (**b**) Effect of facial expressiveness by target gender by target culture on perception of warmth. *Note*. The interaction effects are depicted using the dashed lines and arrows. (**c**) Effect of facial expressiveness by target gender by target culture on perception of overall favorable impression. *Note*. The interaction effects are depicted using the dashed lines and arrows.

To assess the effect of facial expressiveness above and beyond the effect of target gender and/or target culture (Hypothesis 2), we performed three-way mixed model ANOVAs, 2 (facially expressive vs. non-expressive) by 2 (target gender: female vs male) by 2 (target culture: Swiss and Indian), with facial expressiveness as the repeated factor, at the target level (*N* = 159; see Table 2 for the statistical results of the mixed ANOVAs, see Table 3 for the estimated means and standard errors per subgroup, and see Fig. 2 for a visual representation of the results). Adding target gender and target culture as moderators in the statistical models did not change the positive, and large, main effect of facial expressiveness on the outcomes. Accordingly, results showed that expressive targets were perceived as more competent [*F*(1,155) = 404.59, *p* < 0.001, η_p_^2^ = 0.72], warmer [*F*(1,155) = 386.87, *p* < 0.001, η_p_^2^ = 0.71], and made a more favorable overall impression [*F*(1,155) = 721.79, *p* < 0.001, η_p_^2^ = 0.82] than non-expressive targets.

**Table 2:** Results of three-way mixed ANOVA: facial expressiveness by target gender by target culture.

	P. of competence			P. of warmth			P. of overall favorable impression		
	*F*	*p*	*η*^*2*^	*F*	*p*	*η*^*2*^	*F*	*p*	*η*^*2*^
Expressiveness	404.59	0.000	0.72	386.87	0.000	0.71	721.79	0.000	0.82
Gender	5.45	0.021	0.03	12.09	0.001	0.07	5.38	0.022	0.03
Culture	2.43	0.121	0.02	7.55	0.007	0.05	1.05	0.307	0.01
Expressiveness × Gender	0.17	0.679	0.00	0.00	0.963	0.00	0.05	0.823	0.00
Expressiveness × Culture	3.46	0.065	0.02	6.53	0.012	0.04	7.00	0.009	0.04
Gender × Culture	2.08	0.151	0.01	8.69	0.004	0.05	3.40	0.067	0.02
Expressiveness × Gender × Culture	4.26	0.041	0.03	0.32	0.570	0.00	2.45	0.119	0.02

*Notes. N* = 159.

**Table 3:** Estimated means, standard errors, and confidence intervals pertaining to the three-way mixed ANOVA: facial expressiveness by target gender by target culture.

	Perception of competence											
	Swiss						Indian					
	Female			Male			Female			Male		
	*M*	*SE*	95% CI	*M*	*SE*	95% CI	*M*	*SE*	95% CI	*M*	*SE*	95% CI
Expressive	3.59	0.04	[3.52; 3.67]	3.50	0.04	[3.42; 3.58]	3.57	0.07	[3.43; 3.70]	3.51	0.05	[3.41; 3.62]
Non-expressive	2.75	0.04	[2.67; 2.83]	2.77	0.04	[2.69; 2.86]	3.00	0.07	[2.86; 3.14]	2.77	0.06	[2.66; 2.88]

	Perception of warmth											
	Swiss						Indian					
	Female			Male			Female			Male		
	*M*	*SE*	95% CI	*M*	*SE*	95% CI	*M*	*SE*	95% CI	*M*	*SE*	95% CI
Expressive	3.57	0.04	[3.50; 3.65]	3.53	0.04	[3.45; 3.60]	3.69	0.07	[3.56; 3.83]	3.45	0.05	[3.34; 3.55]
Non-expressive	2.75	0.04	[2.67; 2.84]	2.75	0.04	[2.67; 2.84]	3.10	0.08	[2.95; 3.25]	2.82	0.06	[2.70; 2.94]

	Perception of overall favorable impression											
	Swiss						Indian					
	Female			Male			Female			Male		
	*M*	*SE*	95% CI	*M*	*SE*	95% CI	*M*	*SE*	95% CI	*M*	*SE*	95% CI
Expressive	3.67	0.05	[3.58; 3.76]	3.56	0.05	[3.47; 3.66]	3.62	0.08	[3.46; 3.78]	3.47	0.06	[3.34; 3.60]
Non-expressive	2.24	0.05	[2.13; 2.35]	2.29	0.06	[2.18; 2.41]	2.57	0.10	[2.38; 2.77]	2.30	0.08	[2.15; 2.46]

*Notes. N* = 159.

Exploration of the interaction effects including facial expressiveness indicated a three-way interaction effect of facial expressiveness by target gender and by target culture for perception of competence [*F*(1,155) = 4.26, *p* = 0.041, η_p_^2^ = 0.03], a significant two-way interaction effect of facial expressiveness by target culture for perception of warmth [*F*(1,155) = 6.53, *p* = 0.012, η_p_^2^ = 0.04] and for perception of favorable overall impression [*F*(1,155) = 7.00, *p* = 0.009, η_p_^2^ = 0.04].

Concerning *perception of competence*, simple effect analyses indicated that, among facially non-expressive targets, Indian female targets were perceived as more competent than Indian male targets, *M(SE)* = 0.23(0.09); *p* = 0.012; 95% CI = [0.052; 0.411], and that Indian female targets were perceived as more competent than Swiss female targets, *M(SE)* = -0.25(0.08); *p* = 0.002; 95% CI = [− 0.41; − 0.09].

Concerning *perception of warmth* and *perception of overall favorable impression*, simple effect analyses showed that, among non-expressive targets, Indian targets were perceived as warmer and made a more favorable impression than Swiss targets (perception of warmth: *M(SE)* = − 0.20(0.06); *p* < 0.001; 95% CI = [− 0.32; − 0.09]; perception of overall favorable impression: *M(SE)* = − 0.17(0.07);* p* = 0.021; 95% CI = [− 0.32; − 0.03]).

For information purposes only, results also showed a significant main effect of gender for each dependent variable, such that female targets obtained higher scores than male targets and a significant main effect of culture for perception of warmth only, such that Indian targets were perceived as warmer than Swiss targets. See Table 2 for the test statistics.

## Discussion

The goal of this research was to test whether facial expressiveness led to better interview outcomes (observer perception of the targets in terms of competence, warmth, and overall favorable impression) while relying on AI-generated target videos. We used deepfake-based synthesized target videos to manipulate the occurrence of gazing, nodding, and smiling (vs. looking away, no nodding, and no smiling), in a highly standardized manner, in short videos showing the targets reacting to an interview question. Our results showed the positive effect of facial expressiveness on interview outcomes. Accordingly, we found that targets who have a more expressive face (i.e., gazing, nodding, and smiling) were perceived more favorably in terms of competence, warmth, and overall favorable impression than targets who have a less expressive face (i.e., looking away, no nodding and no smiling; Hypothesis 1). Results also indicate that the effect of being facially expressive goes above and beyond target gender and target culture (Hypothesis 2).

Our research contributes to the literature on theoretical, methodological, and practical axes. Concerning the theoretical contributions, our results provide additional evidence of the positive effect of facial expressiveness on interview outcomes. Our results are consistent with research showing that these behaviors positively influence how an individual is perceived by an observer in a job interview setting^23,27–30^. We also show that this effect remains robust as it is stable across culture and gender. Interaction effects regarding culture or gender only occurred for facially non-expressive targets. This suggests that in the absence of meaningful expressive nonverbal facial behavior, judges might fall back on heuristics to evaluate interviewees.

Methodologically, our research provides an alternative to the traditional way of studying the effects of nonverbal behavior. We present a way to rely on a systematic, standardized, and cost-efficient scalable method to develop one’s experimental target video material. This technology-based approach enables researchers to maintain a constant pattern of cues (e.g., nodding when listening) while controlling for or offering sufficient variability of other cues (e.g., target’s appearance) that might affect the studied outcomes. Such levels of standardization and control over key factors (e.g., nonverbal behavior vs. gender, attractiveness, or cultural cues) affecting the outcomes of interest are capital to determine the sole and causal effect of nonverbal behavior on social outcomes. Such an approach might also foster conceptual replications of past studies on nonverbal behavior at a manageable cost. In this vein, thanks to deepfake technologies, researchers could disentangle the effect of nonverbal behavior from surface level diversity markers and their interaction by synthesizing videos of female vs. male targets, or older vs. younger targets. Another example of research related to the manipulation of attractiveness (see^9^ for an example of deepfake-based studies on the effect of facial appearance on teacher evaluations) might include using deepfake to manipulate both nonverbal facial behavior and attractiveness to test the effect of nonverbal charismatic signaling and attractiveness on social influence while the targets give either a charismatic or non-charismatic speech (see^43^ for the original field study). This would allow testing a similar research question using a highly standardized and controlled approach. Because deepfake might also allow to focus on a particular nonverbal behavior cue and allow to control its expression, future research might also focus on isolated nonverbal behavior and manipulate its temporality or expression (rather than focusing on the frequency of smiles, see^35^) while maintaining the expression of nods or gazing constant.

In terms of practical implications, our research reveals the importance of mastering a set of facial expressions to convey a favorable impression during job interviews. We focus on listening behaviors and our research shows that when listening to the recruiter, the applicant has an interest in being expressive and appearing involved. While job interview training focuses mostly on how to convey a good impression when speaking, our results put an emphasis on the rather passive parts of a job interview, namely to convey a good impression when listening to the recruiter. The positive effect of being facially expressive when listening to an interviewer goes above and beyond individual characteristics (i.e., gender and culture) which suggests that job interview training focusing on listening behaviors might be generalized across gender and culture.

### Limitations and future research

One limitation concerns the sample characteristics. The observers came from the same geographical regions (e.g., UK, Ireland). How targets were perceived might thus be typical of these geographical regions, but not of others. Given that the observers came from continental Europe and were asked to judge Indian and European faces, it is plausible that the culture effect is confounded with an in- vs. out-group phenomenon. Moreover, research has shown that a low fit between the target and the judge culture is associated with less favorable outcomes (e.g., liking, hiring outcomes)^44^. Thus, future research could test the effect of facial expressiveness relying on AI-generated videos while using more diverse groups of participants (e.g., targets with varied professional experience and observers from varied geographical origins). This would improve the generalizability of our results and allow testing the idea of cultural fit as a factor influencing the effect of facial expressiveness on interview outcomes.

Relying on deepfake to develop the experimental materials presents its challenges, the first of which is the quality of the videos. This directly depends not only on the quality of the target selfie, but also on the technology itself (i.e., one-shot deepfake). On the one hand, poor quality selfies (e.g., shadows on the face or the slightest facial expression or accessories such as glasses) can create artifacts in the synthesized videos. On the other hand, the technological capabilities affect the quality of the generated material. For instance, given the current state of one-shot deepfake, it was not possible to generate big full-teeth smiles on the target face. Trying to transfer full-teeth smiles from the referent video to the target selfie created unnatural distortion in the target face where fake teeth pierced the target’s lips or appeared on top of the lips. This drastically reduced the realism of the generated target videos. As a solution, we chose to work with subtle smiles (raised lip corners) rather than full-teeth smiles (raised lip corners and showing teeth). We believe that this choice did not reduce the ecological validity of the experimental material because it is probable that people who smile when listening to a job interview question express subtle smiles rather than big smiles. A second challenge is linked to the research question to be addressed. Focusing on facial expressiveness fits well with the use of deepfake. However, researchers interested in both verbal and nonverbal behavior would need to rely on audio-synthesis technology on top of deepfake technologies. In the same vein, researchers interested in nonverbal behaviors expressed in the body or full-body motion would need to rely on posetransfer technologies rather than deepfake (see for example^41^). Posetransfer technologies allow to transfer the motion from a referent video (e.g., a ballet dancer) onto another body (e.g., a target who was videotaped doing another set of motions). In the realm of nonverbal immediacy, posetransfer might be used to study the effect of posture and hand gestures rather than facial expressions.

In both cases (i.e., transfer of facial or body movements), social and behavioral scientists might need to collaborate with computer scientists. On the one hand, an AI-based pipeline might readily be available (e.g., NVIDIA vid2vid toolkit). Even with this off-the-shelf tool, researchers need either sufficient Python programming knowledge to be able to use the toolkit or to collaborate with computer scientists working with it. On the other hand, a readily available toolkit might not correspond to researchers’ needs, it might not exist, or it might not be available for public (noncommercial) use. In this case, the cost of developing customized experimental video material might be too high or again, collaborating with computer scientists could lead to developing the right or custom-made material for a specific research project.

Apart from reproducing and supporting past research showing that being facially expressive when listening to an interview question leads to greater social outcomes, this paper provides a proof-of-concept supporting the use of AI-generated characters, such as deepfake, to develop highly standardized and naturalistic experimental material. The current paper presents an application of one-shot deepfake technology for material development and indicates that relying on such new technologies has potentially a more positive, than negative, effect in the field of research.

## Method

### Material development

One video per target and per condition was generated. Co-authors KS, RS, SSM, and DBJ used machine learning technologies to synthesize the new videos using the target selfie and a set of referent videos showing the combination of nonverbal behaviors specific to each condition.

The original experimental design and protocol for AI-generated video development is based on^18^. The study initially comprised a total of four conditions depending on the combinations of facial cues displayed by the targets: (1) the targets showed gazing at the camera objective as if the target gazed at the interviewer while occasionally nodding and smiling, (2) the targets showed gazing while occasionally nodding, (3) the targets showed gazing only, and (4) the targets showed no gazing, nodding, or smiling when listening to an interview question. All deepfake-based videos were created using the First Order Motion Model—FOMM^40^, which is a motion transfer algorithm that allows users to transfer facial behaviors and associated movements onto a single still image. FOMM requires two essential elements and steps to generate the necessary one-shot deepfake videos. As a first step, it requires a *target input* which consists of a static facial image of individuals (target selfie) with the most neutral facial expression (e.g., closed mouth and no smile). Eye contact with the camera was also required (see “Target procedure for selfie collection” below). The images were then automatically aligned and cropped using the Flickr Faces-HQ—FFHQ^48^ alignment algorithm of the ml4a library to obtain the required images. Images that were not properly aligned were cropped manually.

As a second step, FOMM requires “*referent* *input*” videos showing the required facial expressions that the researchers want to transfer onto the targets’ face using their static facial image (i.e., target selfie). We used an actor to enact specific nonverbal behaviors during an interview. These videos were later used to generate the video stimuli based on a given static image of the target. Examples of target and referent inputs, as well as generated outputs, are provided in Supplementary Material.

#### Target procedure for selfie collection

Target selfies were collected during another study focusing on the meta-perception of competence, warmth, and overall favorable impression. Targets, *N* = 159, aged 18–44, 47.8% women, *M(SD)*_age_ = 23.11(4.11), took part in a two-wave study. During Session 1 (i.e., the pre-selection phase), *targets* first read and signed the consent form to give their informed consent. Second, they completed a questionnaire designed to capture their level of self-efficacy and self-esteem. Third, they reported their socio-demographic information (gender, age, year and faculty of study, country of residence, and ethnic group). Fourth, and finally, they read the instructions to take and send a selfie by email to the lead researchers. The selfie (i.e., target input) was used to synthesize the target videos using one-shot deepfake. During Session 2, the targets watched their own synthesized videos, presented in random order, and for each synthesized video, they reported their meta-perception (i.e., how they think they are perceived by others) on competence, warmth, and overall favorable impression as well as other measures collected for another research project (e.g., feelings toward the version of themselves starred in the synthesized videos and perception of realness, for all videos confounded). Finally, the targets reported their socio-demographics (gender, age, ethnicity, and nationality). The study respects the Declaration of Helsinki and was approved by the Commission for Ethics in Research (CER-HEC).

#### Generated target video

Each generated video lasts around 12 s. Each video shows the target being facially expressive (i.e., gazing, nodding, and smiling) or not (i.e., looking away, not nodding, and not smiling) while seemingly listening to a question asked by a recruiter.

Even if the targets do not speak in the generated videos, an audio track was added to the videos to show targets as interviewees reacting nonverbally when listening to an interview question. To create the audio track, which was the same for all the videos, we asked a research assistant to record himself while uttering an interview question (i.e., “Hello, nice to meet you. I am the HR manager and I will conduct this job interview. To start off, please present yourself and your current situation”). Examples of generated outputs are available under Supplementary Material.

### Sample

We recruited the *observers* on Prolific using gender criteria such that both females and males were equally represented and focusing on United-Kingdom residents. Eight hundred and twenty-three observers, aged 18–89 (50.1% women, *M*_*age*_ = 40.81, *SD* = 14.70) completed the study. Out of the 823 individuals, 94.9% succeeded all the attention checks (3.4% failed one, 1.3% failed two, 0.2% failed three, and 0.1% failed four attention checks). Given the low attention check failure rate and because of discussions on the exclusion of data based on attention check failures (see^45^ for discussion on best practices; see^46^ for “shadow” biases), no participants were excluded. Kindly note that our results virtually remained the same for Hypothesis 1, in case of exclusion of data based on the number of failed attention checks. Because data are aggregated at the target level for Hypothesis 2, we did not test Hypothesis 2 while excluding observers. Table 4 presents a summary of the demographic characteristics of the sample.

**Table 4:** Demographic characteristics of the sample of observers.

	Characteristics	*N* = 823	%
Sex	Women	412	50.1
	Men	410	49.8
	Other	1	0.1
Age	*M* (*SD*)	40.81 (14.70)	
	Min–Max	18–89	
Race	Mixed	23	2.8
	White	750	91.1
	Black or African or African American	10	1.2
	American Indian or Alaska Native	28	3.4
	Asian	1	0.1
	Arab/West Asian	10	1.2
	South East Asian	1	0.1
	Latin	0	0.0
Nationality	UK	773	93.9
	Other	50	6.1
Education level	Some high school, no diploma	46	5.6
	High school degree or equivalent	233	28.3
	Apprenticeship	49	6.0
	Bachelor’s degree (e.g. BA, BS)	333	40.5
	Master’s degree (e.g. MA, MS, MEd)	115	14.0
	Doctorate (e.g. PhD)	23	2.8
	Other	24	2.9
Employment status	Student	65	7.9
	Self-employed	102	12.4
	Unemployed	156	19.0
	Employed full-time	393	47.8
	Employed part-time	107	13.0

The compensation scheme was composed of a fixed fee (£ 1.90 to complete the online study lasting 10 to 15 min) and a performance-based bonus to create an incentive for the task. We attributed a bonus payment of £ 1.00 to the top 20–25% observers who provided the best ratings, that is, the ratings that best mirror the average ratings for the same videos. In the end, 198 observers (24.1%) obtained a bonus payment. We adopted this “compensation game” approach to reduce the risk of bias in responses (see^47^ for justification and application). The study respects the Declaration of Helsinki and was approved by the Commission for Ethics in Research (CER-HEC).

### Design and procedure

In a within-subject experimental design, observers were exposed to one AI-generated video per condition. Each observer saw one video per condition, where targets and conditions were presented in a random order (98.42% of the sample saw different targets across conditions given the technical limitations of using Qualtrics software). Because the targets were recruited from two universities (one in India and one in Switzerland) and given the quantity of data (around 650 AI-generated videos to be combined in the Qualtrics online survey system), data collection was split into three waves. Hence, the video presented for each condition was randomly selected from a pool of 70–75 targets, rather than 200 targets. This also means that observers could randomly watch female or male targets, but only saw targets from a single culture (i.e., the videos seen by observers featured only Swiss or Indian targets).

The observers started the study by reading and completing the consent form to give their informed consent. Then, the observers were informed about the task and the bonus payment scheme: They were to watch the videos, each starring an interviewee (the *target*) listening to a job interview question, and following each video they were to fill in questionnaires. After each video, the observers completed questionnaires to rate how they perceived the target in terms of competence, warmth, hireability, impression, and skills. Additionally, the observers rated the realness of each video. Finally, the observers reported their socio-demographics (gender, age, nationality, education level, employment status, experience in human resources, and knowledge/attitude towards deepfake).

### Measures

We present the dependent variables below. Descriptive statistics and Cronbach’s alpha concerning these variables, at the condition level, are displayed in Table 5.

**Table 5:** Descriptive statistics for the dependent variables, per condition.

	Expressive			Non-expressive		
	*M*	*SD*	α/r	*M*	*SD*	α/r
P. of realness	3.41	0.91	0.89	3.08	1.00	0.91
P. of competence	3.54	0.56	0.85	2.80	0.61	0.83
P. of warmth	3.56	0.58	0.84	2.82	0.65	0.85
P. of overall fav. impression	3.54	0.68	0.93	2.32	0.79	0.95
P. of hireability	3.35	0.72	0.91	2.31	0.80	0.93
P. of impression	3.63	0.76	0.69	2.37	0.87	0.74
P. of skills	3.81	0.74	0.55	2.28	0.90	0.66

*Notes. N* = 823. P., perception.

#### Perception of realness

We used a self-developed 5-item (one reverse-scored) questionnaire to measure the perception of realness of the target videos. A sample item is “the videos seemed realistic.” Observers reported the extent to which they found each video they watched realistic by indicating the extent to which they agree with each item using a 5-point Likert-type scale (1 = *strongly disagree* to 5 = *strongly agree*). We computed the perception of realness by averaging the corresponding items. The higher the score, the more the observers perceived the video as being real.

#### Perception of competence and warmth

We used a 10-item questionnaire based on the Stereotype Content Model^49^. Sample items are “competent” and “friendly.” Observers indicated the extent to which they agreed with each adjective, using a 5-point Likert-type scale (1 = *strongly disagree* to 5 = *strongly agree*). We computed two scores by averaging the corresponding items: perception of competence and of warmth. The higher the score, the more the targets were perceived as competent or warm.

#### Perception of overall favorable impression (hireability, impression, and skills)

For hireability, we used a 4-item questionnaire^50^. A sample item is “As a recruiter, I would be willing to hire the interviewee.” We used an additional self-developed 4-item questionnaire to measure the perception of impression and skills, two items each. Sample items are “the interviewee made a good impression” and “the interviewee showed good interpersonal/soft skills.” Observers reported the extent to which they would be willing to hire the target, perceive them as making a good impression, and appeared skilled, using a 5-point Likert-type scale (1 = *strongly disagree* to 5 = *strongly agree*). The higher the score, the more the observers perceived the targets as hireable, making a good impression, and skilled. Because the three scores are highly correlated (*r*s > 0.70; see Table 1), we created an index of overall favorable impression by averaging the three measures.

### Supplementary Information

Supplementary Information 1.

Supplementary Information 2.

Supplementary Information 3.

Supplementary Information 4.

Supplementary Video 1.

Supplementary Video 2.

Supplementary Video 3.

Supplementary Video 4.

Supplementary Video 5.

Supplementary Video 6.

## Data Availability

The datasets, syntax, data dictionary, and measures presentation for observers are available on OSF repository: https://osf.io/3pavt/?view_only=c06cbb6ff2654ef5a5f299f7e3f87775.
