# Supplementary material for: A deepfake-based study on facial expressiveness and social outcomes
Source: Sci Rep. 2024 Feb 13;14:3642. doi: 10.1038/s41598-024-53475-5 (PMC10864353; doi:10.1038/s41598-024-53475-5)
Supplement: Supplementary file 1 — Supplementary Information 1. [file 41598_2024_53475_MOESM1_ESM.docx]

**Supplementary Material**

The supplementary material contains 9 files. We present below an overview and brief presentation of the files.

| Content | Description | Format | Files |
| --- | --- | --- | --- |
| Appendix | Non parametric tests for Hypothesis 1 | WORD file | 0_Appendix |
| Target input | Target picture (selfie) saved as a .JPG | JPG file | 1_selfie_target1  2_selfie_target2 |
| Referent input | Actor video showing the behaviors to “copy-paste” on the target | MP4 video | 3_referentvideo_expressive  4_referentvideo_unexpressive |
| Output | Generated video based on target and referent inputs | MP4 video | 5_output_target1_expressive  6_output_target1_unexpressive  7_output_target2_expressive  8_output_target2_unexpressive |

*Notes.* Expressive: showing gazing, nodding, and smiling. Unexpressive: not showing gazing, nodding, and smiling.
