# Supplementary material for: A deepfake-based study on facial expressiveness and social outcomes
Source: Sci Rep. 2024 Feb 13;14:3642. doi: 10.1038/s41598-024-53475-5 (PMC10864353; doi:10.1038/s41598-024-53475-5)
Supplement: Supplementary file 2 — Supplementary Information 2. [file 41598_2024_53475_MOESM2_ESM.docx]

# Appendix 1

**Results of non-parametric tests**

We present results of non-parametric tests below because most of the dependent variables were not normally distributed.

## Results of non-parametric tests concerning perception of realness

We performed a one-sample Wilcoxon signed rank test for whether the perception of realness was significantly higher than the scale midpoint. Each pair comprised first a variable corresponding to the score of the perception of realness corresponding to one of the three AI-generated videos, second, a variable consisting of a constant value (=3). Results of Wilcoxon test show that observers perceived the AI-generated videos as realistic (facial expressiveness, *Z* = -11.68, *p* < .001; no facial expressiveness, *Z* = -2.05, *p* = .040).

## Results of non-parametric tests concerning the effect of facial expressiveness on perception of competence, warmth, and overall favorable impression

We performed a paired-sample Wilcoxon signed rank test to test whether the perceptions of competence, warmth, and overall favorable impression were significantly higher in the facially expressive condition compared to the unexpressive condition. Results show that the perceptions of competence, warmth, and overall favorable impression were more positive for more facially expressive targets (competence: *Z* = -19.80, *p* < .001; warmth: *Z* = -19.29, *p* < .001; and overall favorable impression: *Z* = -21.95, *p* < .001).
