# Supplementary figures and images for: A deepfake-based study on facial expressiveness and social outcomes
Source: Sci Rep. 2024 Feb 13;14:3642. doi: 10.1038/s41598-024-53475-5 (PMC10864353; doi:10.1038/s41598-024-53475-5)

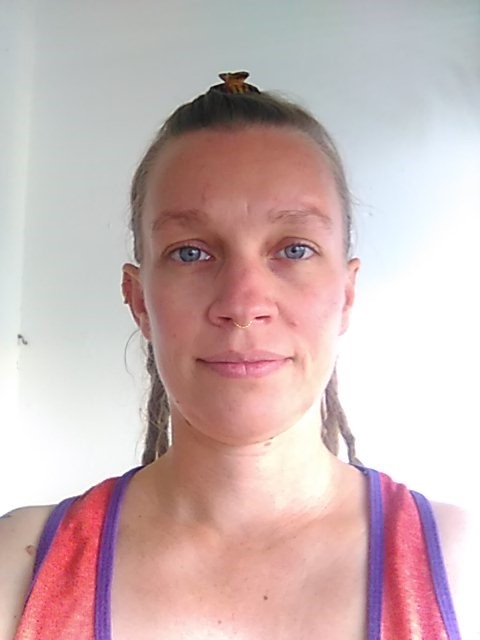

Supplement: Supplementary file 3 — Supplementary Information 3. [file 41598_2024_53475_MOESM3_ESM.jpg]

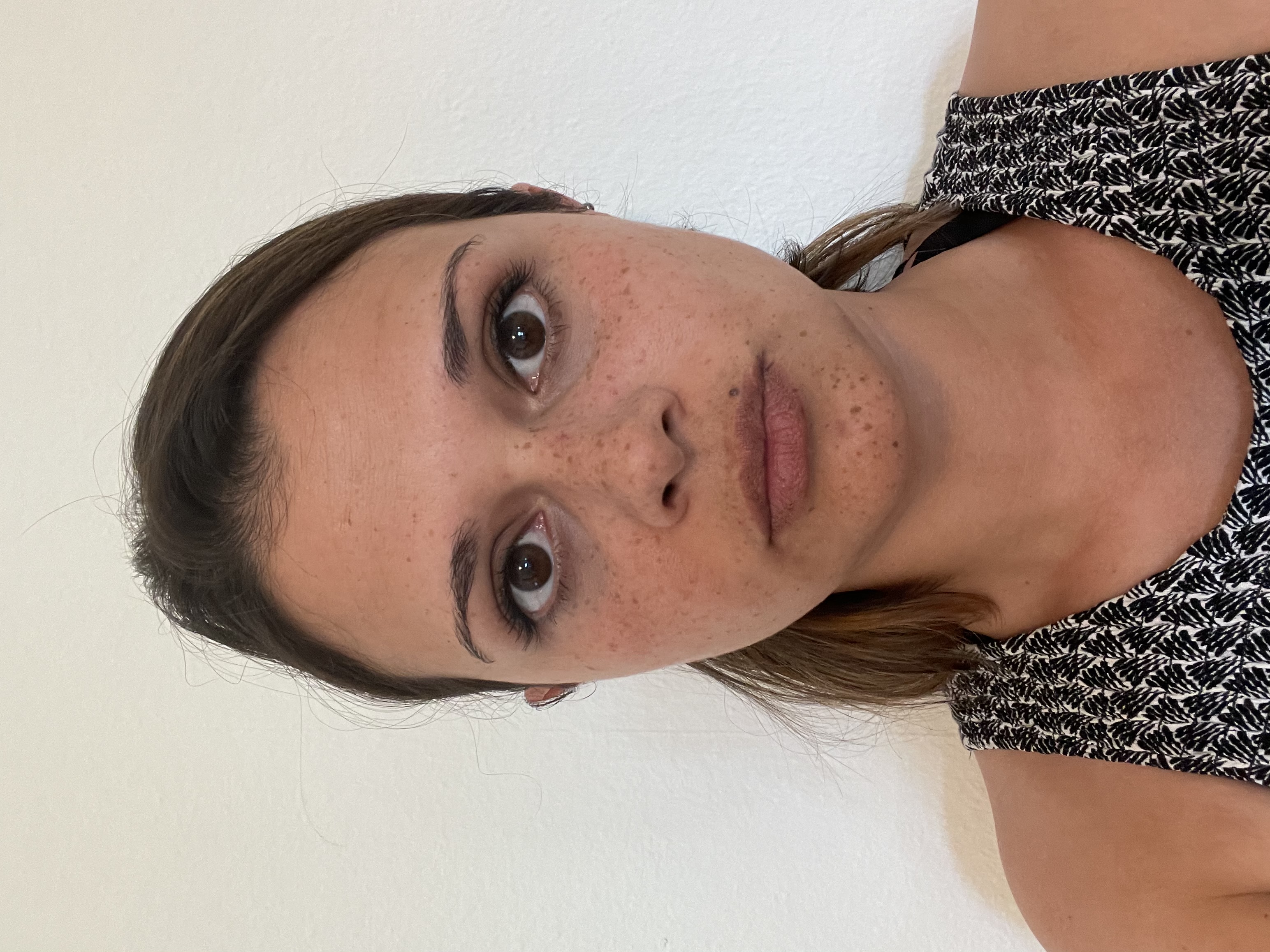

Supplement: Supplementary file 4 — Supplementary Information 4. [file 41598_2024_53475_MOESM4_ESM.jpeg]
